# Supplementary material for: Prior salpingectomy impairs the retrieved oocyte number in in vitro fertilization cycles of women under 35 years old without optimal ovarian reserve
Source: PLoS One. 2022 May 4;17(5):e0268021. doi: 10.1371/journal.pone.0268021 (PMC9067640; doi:10.1371/journal.pone.0268021)
Supplement: S1 File — (DOCX) [file pone.0268021.s001.docx]

The total number of retrieved oocytes in IVF

| Fig. # | mean | standard deviation |
| --- | --- | --- |
| **Fig. 1A** |  |  |
| Study | 10.4 | 5.2 |
| Control | 12.2 | 3.8 |
| **Fig. 1B** |  |  |
| Study (AMH<4) | 6.4 | 3.1 |
| Control (AMH<4) | 9.9 | 3.0 |
| **Fig. 1C** |  |  |
| Study (AMH≥4) | 13.1 | 4.8 |
| Control (AMH≥4) | 12.8 | 3.7 |
| **Fig. 2A** |  |  |
| Hydrosalpinx | 10.3 | 6.2 |
| Ectopic pregnancy | 10.5 | 3.9 |
| Control | 12.2 | 3.8 |
| **Fig. 2B** |  |  |
| Hydrosalpinx (AMH<4) | 6.0 | 3.4 |
| Ectopic pregnancy (AMH<4) | 6.9 | 2.9 |
| Control (AMH<4) | 9.9 | 3.0 |
| **Fig. 2C** |  |  |
| Hydrosalpinx (AMH≥4) | 13.3 | 5.8 |
| Ectopic pregnancy (AMH≥4) | 12.8 | 3.7 |
| Control (AMH≥4) | 12.8 | 3.7 |
| **Fig. 3A** |  |  |
| Bilateral | 10.6 | 5.8 |
| Unilateral | 10.3 | 4.9 |
| Control | 12.2 | 3.8 |
| **Fig. 3B** |  |  |
| Bilateral (AMH<4) | 5.3 | 1.5 |
| Unilateral (AMH<4) | 6.7 | 3.3 |
| Control (AMH<4) | 9.9 | 3.0 |
| **Fig. 3C** |  |  |
| Bilateral (AMH≥4) | 11.3 | 5.1 |
| Unilateral (AMH≥4) | 14.3 | 4.4 |
| Control (AMH≥4) | 12.8 | 3.7 |

The total number of retrieved oocytes in IVF

| Fig. # | # numbers | Min | Q1 | Median | Q3 | Max |
| --- | --- | --- | --- | --- | --- | --- |
| **Fig. 1A** |  |  |  |  |  |  |
| Study | 54 | 2 | 7 | 9 | 13 | 24 |
| Control | 59 | 6 | 9 | 12 | 14.5 | 21 |
| **Fig. 1B** |  |  |  |  |  |  |
| Study (AMH<4) | 14 | 2 | 4.25 | 7 | 7.75 | 12 |
| Control (AMH<4) | 10 | 6 | 8 | 9.5 | 10.75 | 17 |
| **Fig. 1C** |  |  |  |  |  |  |
| Study (AMH≥4) | 25 | 6 | 8 | 13 | 17 | 22 |
| Control (AMH≥4) | 39 | 6 | 10 | 13 | 15.5 | 21 |
| **Fig. 2A** |  |  |  |  |  |  |
| Hydrosalpinx | 28 | 2 | 5.75 | 8 | 13.25 | 24 |
| Ectopic pregnancy | 26 | 2 | 7 | 10 | 13 | 18 |
| Control | 59 | 6 | 9 | 12 | 14.5 | 21 |
| **Fig. 2B** |  |  |  |  |  |  |
| Hydrosalpinx (AMH<4) | 7 | 2 | 3.5 | 5 | 8.5 | 11 |
| Ectopic pregnancy (AMH<4) | 7 | 2 | 6.5 | 7 | 7 | 12 |
| Control (AMH<4) | 10 | 6 | 8 | 9.5 | 10.75 | 17 |
| **Fig. 2C** |  |  |  |  |  |  |
| Hydrosalpinx (AMH≥4) | 13 | 6 | 8 | 13 | 20 | 22 |
| Ectopic pregnancy (AMH≥4) | 12 | 7 | 10.5 | 13 | 14.75 | 18 |
| Control (AMH≥4) | 39 | 6 | 10 | 13 | 15.5 | 21 |
| **Fig. 3A** |  |  |  |  |  |  |
| Bilateral | 17 | 4 | 7 | 8 | 13 | 24 |
| Unilateral | 37 | 2 | 7 | 10 | 13 | 22 |
| Control | 59 | 6 | 9 | 12 | 14.5 | 21 |
| **Fig. 3B** |  |  |  |  |  |  |
| Bilateral (AMH<4) | 3 | 4 | 4.5 | 5 | 6 | 7 |
| Unilateral (AMH<4) | 11 | 2 | 4.5 | 7 | 8.5 | 12 |
| Control (AMH<4) | 10 | 6 | 8 | 9.5 | 10.75 | 17 |
| **Fig. 3C** |  |  |  |  |  |  |
| Bilateral (AMH≥4) | 10 | 6 | 7.25 | 9.5 | 15 | 20 |
| Unilateral (AMH≥4) | 15 | 7 | 12.5 | 14 | 17.5 | 22 |
| Control (AMH≥4) | 39 | 6 | 10 | 13 | 15.5 | 21 |
