# Supplementary material for: Prior salpingectomy impairs the retrieved oocyte number in in vitro fertilization cycles of women under 35 years old without optimal ovarian reserve
Source: PLoS One. 2022 May 4;17(5):e0268021. doi: 10.1371/journal.pone.0268021 (PMC9067640; doi:10.1371/journal.pone.0268021)
Supplement: Attachment — Submitted filename: review report.docx [file pone.0268021.s002.docx]

Dear Editor

Thank you very much for the opportunity to evaluate this article. The subject of the study is current. A clear, simple and understandable writing language was used in the article. I believe it will benefit the reader. This is a good study comparing the number of oocytes collected after salpingectomy. Congratulations to the authors.

Below are my recommendations for the study.

Best regards

- Some english grammar mistakes should be corrected.

-I suggest that some studies comparing the histopathological effects of salpingectomy and proximal tubal occlusion procedures on ovarian tissue should be given as examples [e.g. *Atilgan R, Pala Ş, Kuloğlu T, Şanli C, Yavuzkir Ş, Özkan ZS. Comparison of the efficacy between bilateral proximal tubal occlusion and total salpingectomy on ovarian reserve and the cholinergic system: an experimental study. Turk J Med Sci. 2020 Jun 23;50(4):1097-1105. doi: 10.3906/sag-2002-179.*

*Atilgan R, Kuloğlu T, Boztosun A, Orak U, Baspinar M, Can B, Sapmaz E. Investigation of the effects of unilateral total salpingectomy on ovarian proliferating cell nuclear antigen and follicular reserve: experimental study. Eur J Obstet Gynecol Reprod Biol. 2015 May;188:56-60. doi: 10.1016/j.ejogrb.2015.02.028*.]
