# Supplementary material for: Prior salpingectomy impairs the retrieved oocyte number in in vitro fertilization cycles of women under 35 years old without optimal ovarian reserve
Source: PLoS One. 2022 May 4;17(5):e0268021. doi: 10.1371/journal.pone.0268021 (PMC9067640; doi:10.1371/journal.pone.0268021)
Supplement: Attachment — Submitted filename: Response to Reviewers.docx [file pone.0268021.s004.docx]

**Comment Responses and Rebuttal**

Dear Academic editor:

We greatly appreciated the valuable comments for improving our study. We have revised our manuscript accordingly and for adherence to the journal requirements. Our point-by-point responses to your comments are as follows.

**# Academic Editor Comments**

Although the paper has been improved, there are still some problems that need to be addressed:

**Comment 1**. The Authors stated in their response ‘the effect of salpingectomy history was no longer significant after applying the full model of multivariate analysis that included adjustment for AMH level and AFC’ This is a strong message that should be clear in the Abstract.

**Response 1:** Indeed, this message should be clear, and we have added in the Abstract, subtitled “measurements and main results”, line 64-69. Also, we have modified the context of the “conclusion” section in the Abstract with highlighting the message as stated above.

The problem that univariate regression analysis in our study indicated that factors such as salpingectomy history, AMH, and AFC were all significantly associated with the number of retrieved oocytes. However, in the multivariate-adjusted complete model, only AFC and AMH levels continued to exhibit significant associations with the number of retrieved oocytes. The explanation is that the effect of previous salpingectomy toward ovarian response are not as direct as ovarian surrogate biomarkers, which had been collected just around the time of controlled ovarian stimulation and transvaginal oocytes retrieval.

Salpingectomy impaired the circulation of ovary and may cause ischemic-reperfusion injury, and indirectly affect the ovarian function and response to fertility drugs afterwards. Therefore, in the full model of multivariate analysis, we can see that AFC and AMH have more direct influences toward number of retrieved oocytes than other factors.

**Comment 2**. The abstract needs to be improved as the message provided is misleading.

**Response 2:** Thank you for the comments and suggestions. We have added the important message of our statistical results in the Abstract, subtitled” measurements and main results” and “conclusion”. Although the full mode of multivariate analysis did not reveal the negative impact of previous salpingectomy on the number of retrieved oocytes, the detrimental effect to the ovarian function and response still presented via the decreased ovarian surrogate markers (AFC and AMH). Therefore, infertile patients with previous salpingectomies and suboptimal ovarian reserve should be paid attention to their ovarian response, however, postsurgical AMH and AFC levels still possess a more direct predictive value on ovarian response than the history of salpingectomy.

**Comment 3.** Figure legends are missing. How are data presented in the bars?

**Response 3:** We do apologize for the mistakes of incomplete figure legends. According to the policy of PLOS ONE journal for figure legends, we inserted the figure legends (including Fig. 1 to 3) by the way of inlay in the Manuscript, page 14 and 16. The data in the bars, including the values of means, standard deviations, were provided in the minimal data set of supplementary files. P values presented as an actual number in the figure above the bar plot when there was a significant difference.

**Comment 4.** The choice of limits of AMH (4 ng/mL) and AFC (12) for the subgroup analysis needs to be justified.

**Response 4:** In the full mode of multivariate analysis within our study, we found that AMH and AFC continued to be significantly associated with the number of retrieved oocytes. We further divided the subgroups with different level of surrogate ovarian biomarkers to realize the impact on ovarian response.

The cutoff value for AMH (4 ng/mL) was based on the reported 75th percentile of AMH levels of generally healthy Chinese women aged 35 years by the reference [21], BJOG 2020;127(6):720-8. The 75^th^ percentile of AMH levels between 35 to 36 year-old chinese women lies between 4.229 to 3.973 ng/mL, which can be found in the Table 1 of the reference. Another reason for the cutoff value is because the limited case number in our study, we chose accordingly rather than calculated median AMH of our study. The cutoff value for AFC (12 follicles) was based on the calculated median AFC of all enrolled patients in our study. Above description was written in the Materials and Methods, section “Controlled ovarian stimulation and outcome measures” last two sentences.

The reason we used subgroup analysis to distinguish the patients with optimal ovarian reserve, which can be normal or hyper-responders and often with plenty number of retrieved oocytes and could mask the effect of history of salpingectomy.
